# The Natural Dentine for Capping Exposed Pulps

**Published:** 1880-06

**Authors:** Thomas C. Stellwagen


### ARTICLE IX.

### *The Natural Dentine for Capping Exposed Teeth Pulps.*

BY DR. THOMAS C. STELLWAGEN.

Some time since I called the attention of the dental and medical professions to the use of the actual cautery, by means of a coal of fire made by igniting hickory pivot wood, such as is prepared for dentists by being seasoned and condensed by passing through steel draw-plates.

It is recommended by its simplicity, inexpensiveness and efficiency. Use has proven it of value in treating superficial abnormal growths, hæmorrhage, sensitive dentine, etc.

To-day I ask you to consider the natural dentine for capping exposed teeth pulps.

It was during the examination of the last class but one that graduated from the Philadelphia Dental College that it occurred to me to seek for some more natural and simple means for the immediate covering of exposed dental pulp tissue.

The number of articles, already used for the purpose is so great, that the mere attempt to recall them requires considerable mental effort. I am about to add one more with the hope of shortening the list by having it universally adopted, to the exclusion of the others.

Medical science has demonstrated that where an ulceration of the skin has become chronic, the transplantation of small particles of epidermis from a healthy surface of the body, has the effect of assisting in the healing of the ulceration; the transplanted tissue cells acting as nucleuses about which the new first develop, as coral reefs in the ocean eventually become islands. A step further and we remember that small portions of bone or periosteum are often used in the same manner for producing centers of ossification, where bony tissue has been lost. They seem to resemble the points of ossification in the cartilaginous skeleton of the infant.

Attempts to assimilate the capping material to the dental substance have been made which are so ingenious and useful that it would seem to be a pity that they had not realized the hopes of their projectors. They serve, however, to show that all who study the operation wish to attain the greatest similarity of tissue.

Why not then use the tooth substance?

After removing the diseased structure, if the pulp is exposed allow the wounded surface to glaze over, and then, with a keen, sharp excavator, shave off from the walls of the cavity enough healthy dentine to cover over the point to be capped. After this is accomplished place a small metallic cap—platina I generally prefer—to which enough gutta-percha has been attached, by warming, to hold it in position, and proceed to fill, taking care not to displace this bridge, which is designed to protect from pressure and as an additional non-conductor of heat and electrical currents.

On no account wash or remove the powdered healthy dentine from the surface of the glazed pulp. Keep all other substances from coming in contact with this delicate tissue, whether they be the so-called medicaments such as creosote, carbolic acid, chloride of zinc or even simple alcohol or water; they are foreign bodies and are apt to excite inflammation.

The operation as described has been most successful with me, and for about eighteen months I believe I have capped every exposed pulp this way. My successes embolden me to beg of you to accept my recommendation of it as worthy of trial.—*Trans. American Dental Association.*

---

